# Stenotrophomonas maltophilia Bloodstream Infection Outbreak in an Acute Care Hospital — Alameda County, California 2022–2023

**DOI:** 10.1017/ash.2024.280

**Published:** 2024-09-16

**Authors:** Sana Khan, Axel Vazquez Deida, Steven Langerman, Jennifer C. Hunter, Alison Halpin, Alyssa Kent, Paige Gable, Frances Knight, Amit Chitnis, Eileen Dunne, Kiran Perkins, Dustin Heaton, Munira Shemsu, Margarita Elsa Villarino, Kavita Trivedi, Jeffrey Silvers

**Affiliations:** Centers for Disease Control and Prevention; Alameda County Public Health; Healthcare Associated Infections (HAI) Program, CDPH; Alameda County Public Health Department; Sutter Health

## Abstract

**Background:** Stenotrophomonas maltophilia is an opportunistic pathogen found in healthcare settings. During April–September 2022, nine S. maltophilia bloodstream infections (BSIs) were identified among intensive care unit (ICU) patients at a hospital in Alameda County, California. Whole genome sequencing found isolates to be highly related. Despite implementation of infection prevention and control (IPC) interventions, four additional S. maltophilia BSIs were identified during June–September 2023. We investigated to identify risk factors for infection and stop transmission. **Methods:** We conducted a matched case-control study. A case was defined as S. maltophilia isolated from a blood culture from an ICU patient with a fever during April 2022–September 2023; control-patient subjects were patients admitted to the ICU during the same period with hospital stay greater than or equal to their matched case. Three control subjects were matched to each case. We extracted information on risk factors for infection from medical charts and observed IPC practices in hospital locations of interest. We collected environmental samples from the ICU, radiology unit, and emergency department. **Results:** Among 13 cases and 39 control subjects, patients exposed to iodinated contrast Omnipaque-300 (odds ratio [OR]: 5.7; 95% CI: 1.2–28.0), injectable propofol (OR: 12.2; 95% CI: 1.5–101.4), or fentanyl (OR: 9.2; 95% CI: 1.8–Inf.) were more likely to have a S. maltophilia BSI, compared with control-subjects. IPC deficiencies included improper cleaning and storage of medical equipment, including the contrast injection system, and patient care supplies. The outbreak strain of S. maltophilia was not isolated from environmental samples. **Conclusions:** Although a point-source was not identified, S. maltophilia was likely transmitted through improper IPC practices involving injectable contrast or anesthesia. Recommendations on proper cleaning and disinfection of the contrast injection system and proper storage, preparation, and administration of medications were made to reduce risk for contamination.

